# Surgical Treatment of Femoral Neck Fractures: A Brief Review

**DOI:** 10.3390/geriatrics5020022

**Published:** 2020-04-01

**Authors:** Ellen Lutnick, Jeansol Kang, David M. Freccero

**Affiliations:** 1 Jacobs School of Medicine and Biomedical Sciences, 955 Main St., Buffalo, NY 14203, USA; ellenlut@buffalo.edu; 2 Boston University School of Medicine, Department of Orthopaedics, 850 Harrison Ave. Dowling 2N, Boston, MA 02118, USA; jeansol.kang@bmc.org

**Keywords:** hip fracture, femoral neck fractures, internal fixation, hemiarthroplasty, total hip arthroplasty, cemented, uncemented

## Abstract

Hip fracture is a cause for concern in the geriatric population. It is one of the leading causes of traumatic injury in this demographic and correlates to a higher risk of all-cause morbidity and mortality. The Garden classification of femoral neck fractures (FNF) dictates treatment via internal fixation or hip replacement, including hemiarthroplasty or total hip arthroplasty. This review summarizes existing literature that has explored the difference in outcomes between internal fixation, hemiarthroplasty, and total hip arthroplasty for nondisplaced and displaced FNF in the geriatric population, and more specifically highlights the risks and benefits of a cemented vs. uncemented approach to hemiarthroplasty.

## 1. Introduction

The geriatric population, defined as those aged 65 and older, is the fastest growing demographic in the world. Hip fracture, including femoral neck fracture (FNF), is a cause for concern in this group, as they are one of the most common traumatic injuries in elderly patients and are associated with high rates of mortality and functional loss [1,2,3].

In general, hip fractures most commonly occur in elderly female patients, particularly in white populations of North America and Europe, and the incidence increases exponentially with age [4]. Well-established nonmodifiable risk factors for sustaining hip fractures include female sex, increasing age, ethnic origin, and family history of osteoporotic fragility fractures. Modifiable or lifestyle risk factors include low body mass index (<18.5), smoking, alcohol abuse, poor nutritional status, and low levels of baseline physical activity [5]. Other risk factors are often related to aging, which, in turn, are usually associated with increased risk of falls—these include muscle weakness, deficits in balance or coordination, deteriorating eyesight, and medication side effects.

Prior epidemiological studies have predicted that as the geriatric population increases, the hip fracture incidence will increase as well. From 1986 to 1995, the average annual incidence was approximately 950 per 100,000 for women and 415 per 100,000 for men, with greater increases seen for individuals over age 75 than for those ages 65 to 74, and the worldwide incidence was estimated to be over six million by 2050 [2,6]. However, more recent evidence has suggested that the incidence may have reached a plateau. Some population-based studies from North America and Europe have even reported decreases in the number of hip fractures [4,7,8,9]. Despite this, it is likely that hip fracture treatment will continue to comprise a large part of the orthopedic surgeon’s workload.

The ultimate goal of treatment for these hip fractures is to allow early mobilization, which often necessitates surgical procedures to fix or replace the joint. Numerous studies have been undertaken over the decades to determine the optimal intervention to improve patient outcomes and decrease mortality. The objective of this review was to evaluate the existing literature and elucidate the advantages and disadvantages associated with different surgical treatment options available for the various types of FNF, particularly displaced FNFs. The risks associated with cemented vs. uncemented arthroplasty were also explored. Specific consideration was also given to bone cement implantation syndrome.

## 2. Results

### 2.1. Classification of Femoral Neck Fractures

**Figure 1:**
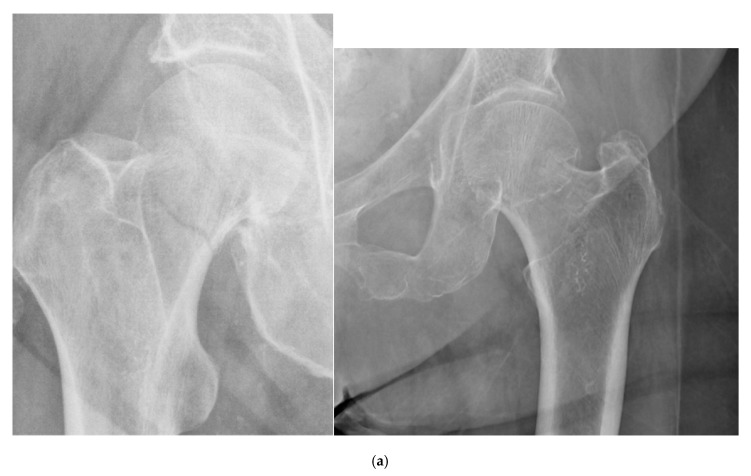

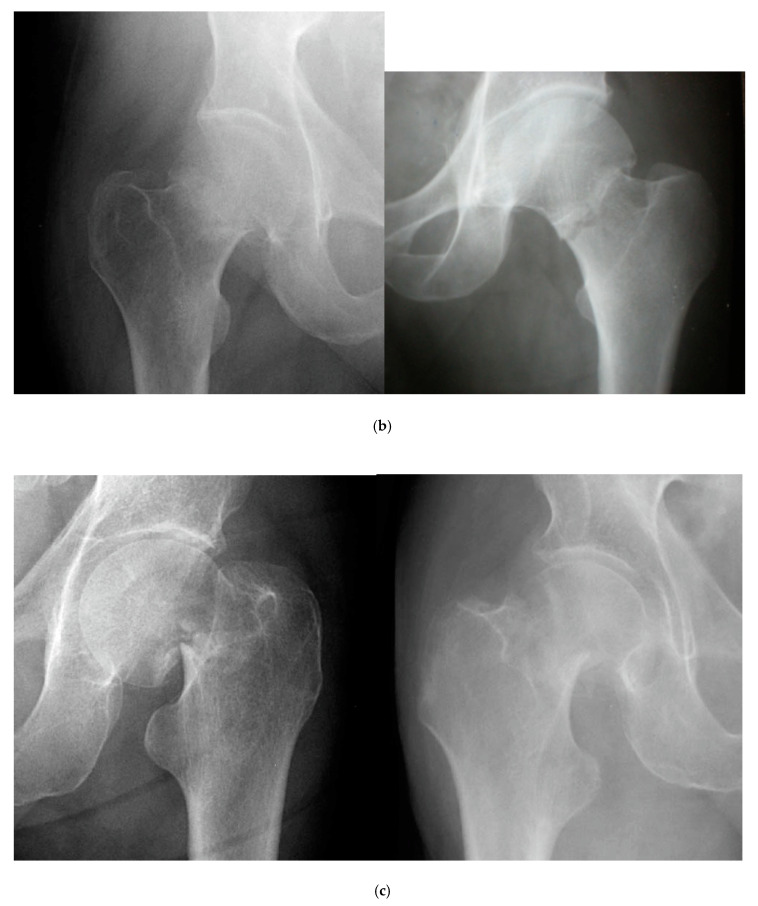

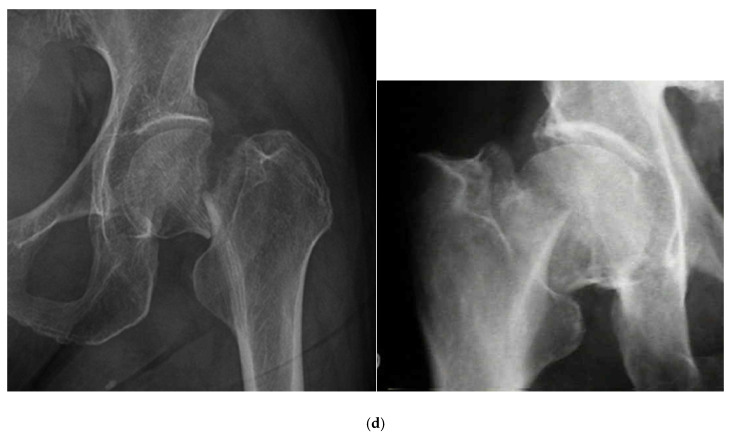
Example radiographs demonstrating Garden classification: (**a**) Garden I; (**b**) Garden II; (**c**) Garden III; (**d**) Garden IV.

One of the most commonly used classification systems for femoral neck fractures is the Garden classification, which was originally described in 1961. This system comprises four stages, which are based on the degree of fracture displacement as seen on anteroposterior (AP) radiographs [10] (Figure 1):

- Stage I: incomplete fracture; nondisplaced, valgus impacted
- Stage II: complete fracture; nondisplaced
- Stage III: complete fracture; partially displaced
- Stage IV: complete fracture; fully displaced

Garden type I fractures involve a lateral fracture line that does not cross the medial cortex, and are thus considered incomplete fractures. Garden type II fractures are complete, but with minimal to no displacement. In Garden type III fractures, the displacement is such that the femoral head is still in some contact with the femoral neck. Garden type IV fractures are completely displaced, and there is no contact between the femoral head and femoral neck. These four stages, which can also be simplified into nondisplaced (types I and II) and displaced (types III and IV), rank the fracture from most to least stable and are often used to direct the approach to treatment. Although interobserver agreement across all four grades has been shown to be relatively poor, especially between types I and II and between types III and IV, much higher levels of agreement have been demonstrated in determining whether fractures were nondisplaced or displaced [10,11]. 

### 2.2. Nondisplaced Femoral Neck Fractures: Internal Fixation vs. Arthroplasty

**Figure 2:**
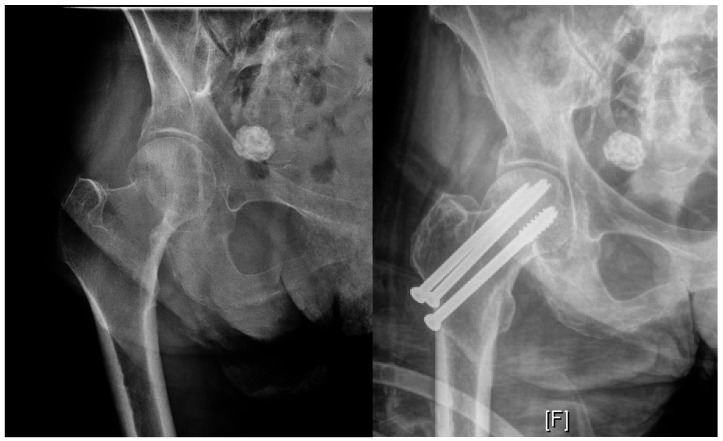
Example radiographs demonstrating internal fixation for Garden I (Left: Preoperative, right: Postoperative).

Nondisplaced Garden I and II fractures are treated with fixation in most patients (Figure 2). Contemporary fixation techniques include either a cannulated screw system, which involves placing 2–4 screws across the fracture into the femoral head, or a sliding hip screw device, where a lag screw across the fracture slides along the barrel of a side plate that is fixed into the proximal femur. Cannulated screws can often be placed percutaneously, and both techniques can be performed with minimal surgical exposure if open. This allows for short operative times, relatively little blood loss, and fewer immediate postsurgical complications compared to arthroplasty techniques [12,13,14,15].

Several studies have compared cannulated screw fixation with sliding hip screw devices for the treatment of nondisplaced FNFs, with the general consensus that both procedures yield equivalent results. A meta-analysis of over 25 trials by Parker et al. found no clear evidence for the superiority of one fixation technique over the other based on the outcome parameters of fracture healing complications, reoperations, or mortality. However, the analysis may have been clouded by the heterogeneity of reporting outcomes in the included studies [16]. Of note, the use of sliding hip screws was reported to have longer operative times and higher blood loss compared to screw fixation.

More recently, a large, international, multicenter randomized controlled trial (RCT) known as the FAITH trial compared cannulated screws to sliding hip screw in 1108 patients [17]. Although this trial included both nondisplaced and displaced fractures, only patients who deemed fixation as their treatment of choice were included. There were no significant differences between fixation techniques in terms of the primary study endpoint of hip reoperation within 24 months, as well as for nonunion, implant failure, infection, or fracture shortening. The authors concluded that the sliding hip screw did not confer any benefit over screw fixation in terms of reoperation.

Overall, internal fixation of nondisplaced FNFs that heal without complications usually confer acceptable functional outcomes, with most patients returning to their previous level of mobility. Healed fractures that still present problems are often related to femoral neck collapse or shortening, which can adversely affect hip abductor muscle function and gait. Recent data from a retrospective case series show that this is a significant risk with internal fixation—of 130 patients with Garden I and II fractures treated with percutaneous pinning, 42% of Garden I and 63% of Garden II fractures demonstrated >10-mm collapse at 12-month follow-up [18]. Studies have shown this to be associated with worse overall outcomes based on Harris hip scores, SF-36, and EuroQol disability questionnaires, and it is often a cause for reoperation [19,20]. This association could be seen even with a shortening of 5 mm, but was particularly pronounced when it was greater than 10 mm. Other causes for reoperation after internal fixation are generally related to complications with fracture healing, such as avascular necrosis (AVN) or nonunion, which occur at rates of approximately 6% and 7%, respectively [12,13,21]. The overall reoperation rate for all causes in nondisplaced fractures is around 14% [17].

In the face of fracture healing complications after internal fixation, arthroplasty for nondisplaced FNFs may be considered. Parker et al. compared 346 patients with nondisplaced FNF treated with internal fixation and 346 matched patients with displaced FNF treated with hemiarthroplasty (HA) [15]. They reported a higher reoperation rate for the fixation group (43/346 vs. 14/346 for HA), but significantly lower one-year mortality (19% vs. 26% for HA), better mobility and less pain at one year, shorter operative times and hospital stays, and lower rates of perioperative complications. However, these results should be interpreted with caution, as they assume that patients with nondisplaced and displaced FNF are similar and comparable. A more recent RCT by Dolatowski et al. compared screw fixation and hemiarthroplasty solely for nondisplaced FNF in 219 patients over the age of 70 [22]. At the two-year follow-up, there was no significant difference between groups in terms of hip function (as measured by Harris hip scores) or hip pain. However, the hemiarthroplasty group displayed better mobility as evaluated by the Timed Up and Go (TUG) test and had significantly lower risk for major reoperation (5/108 vs. 22/110 for screw fixation). These findings suggest that although hemiarthroplasty is not superior to screw fixation in terms of functional outcomes, it may be beneficial for certain populations with lower physiological reserves who may benefit from early mobilization and may not tolerate secondary procedures.

### 2.3. Displaced Femoral Neck Fractures: Internal Fixation vs. Arthroplasty

Treatment methods for displaced Garden III and IV fractures have historically been much more variable than those for nondisplaced fractures. Whereas the tendency is for younger patients (age less than 60) to be treated with fixation and older patients (age greater than 80) to be treated with arthroplasty, there is a relative gray area for patients between the ages of 60 and 80 who may be treated with fixation, hemiarthroplasty, or total hip arthroplasty (THA).

Arthroplasty options, as mentioned above, remove the risks of nonunion and AVN that may be seen with internal fixation. On the other hand, they introduce the risks of increased operative time and blood loss, dislocation, hardware loosening, increased infection rates, and acetabular cartilage erosion [23]. Several prospective RCTs have shown arthroplasty to yield superior results compared to internal fixation for displaced FNF in terms of treatment failure/reoperation rates and functional outcomes [23,24,25,26,27,28]. Rogmark et al. conducted a multicenter study of 409 patients in Sweden with Garden III or IV FNF randomized to internal fixation or arthroplasty [28]. At two-year follow-up, the authors found a failure rate of 43% in the internal fixation group compared to 6% in the arthroplasty group, as well as significant differences in impaired walking and severe pain favoring the arthroplasty group. At 10-year follow-up, differences in failure rate remained statistically significant, with 45.6% in the internal fixation group and 8.8% in the arthroplasty group, but there were no differences in mortality or patient-reported outcomes, including hip pain while walking, reduced mobility, and the need for walking aids [27]. Similarly, Keating et al. compared internal fixation with arthroplasty (including HA and THA) in a multicenter RCT of 207 patients age 60 or greater [26]. At two-year follow-up, 39% of patients in the internal fixation group required secondary surgery, compared with only 5% in the hemiarthroplasty group and 9% in the THA group. Patients who had undergone internal fixation also reported the poorest outcomes in terms of functional and quality of life scores. Data from other RCTs were summarized in a 2006 Cochrane review by Parker et al., which included 17 trials comparing internal fixation and arthroplasty for displaced FNF [23]. The combined data from 2694 patients demonstrated higher rates of deep infection, blood loss, and transfusions associated with arthroplasty, but once again emphasized the significantly lower rates of reoperation. This finding has been corroborated by more recent studies, which also support improved functional outcomes with arthroplasty in both short- and long-term follow-up periods [24,25].

The significance of failure and reoperation rates are highlighted by a case-control study of 214 patients conducted by McKinley et al., which matched 107 patients who underwent early salvage cemented THA following failed internal fixation of a Garden III or IV FNF with 107 age- and gender-matched patients who received a primary cemented THA for the same fracture [29]. The conversion THA group reported significantly more early complications, including superficial infections and dislocations, compared to the primary THA group, as well as significantly worse functional outcomes at the one-year and final follow-up time points. These results suggest that THA may be favorable as an initial treatment, especially in elderly, frail patients who may not be able to tolerate revision procedures.

### 2.4. Displaced Femoral Neck Fractures: Hemiarthroplasty vs. Total Hip Arthroplasty

**Figure 3:**
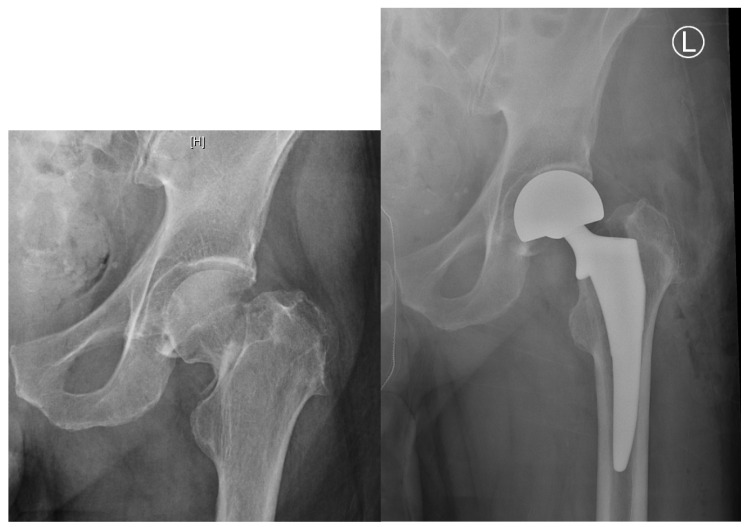
Example radiographs demonstrating total hip arthroplasty Garden III fracture (Left: Preoperative, right: Postoperative).

Despite general consensus regarding the advantage of arthroplasty over internal fixation for displaced fractures, the choice between HA and THA for elderly patients remains controversial (Figure 3). Previous data have suggested that HA may be sufficient for low-demand patients with numerous comorbidities, while THA may produce better functional outcomes, especially for more active, independent patients. However, recent studies continue to produce conflicting evidence [26,30,31,32,33,34,35,36,37]. Despite the lack of clear supporting evidence, recent reports on the trends in treatment in the United States show that although the majority of patients with FNF receive HA, the overall use of HA has declined over the past few decades while the use of THA has increased [38].

Keating et al. reported on the functional and quality of life outcomes in previously independent patients who were randomized to receive THA or HA for displaced FNF [26]. At 24-month follow-up, the THA group described significantly better scores on the Hip Rating questionnaire compared to the hemiarthroplasty group, particularly in the walking and function subscores. Of note, planned subgroup analysis showed that these improved results with THA were more noticeable in patients aged 60 to 74 than those aged 75 and older. Similarly, in a RCT of 81 patients with displaced FNF (mean age 75) who were randomized to hemiarthroplasty or THA, Baker et al. found that patients who received THA were able to walk farther and had better functional outcomes via Oxford Hip scores at a mean follow-up of three years [30]. All patients in the study had been mobile and independent prior to injury. The authors concluded that THA provided better short-term clinical results with fewer complications compared to hemiarthroplasty.

Several other studies have further supported the advantage of THA over HA for displaced FNF [33,34,36], while others have demonstrated equivalent results for functional outcomes, reoperations, and mortality [31,32,35,37,39]. Van den Bekerom et al. reported on a RCT of 252 patients with a mean age of 81 years, and found no differences in modified Harris hip scores, revision rate, or mortality rate between HA and THA at one- and five-year follow-up [39]. However, based on the finding of significantly longer operative times, higher blood loss, and higher dislocation rates (8/115 vs. 0/137 for HA) in the THA group, the authors recommended against the use of THA in patients older than 70. More recently, a RCT of 120 octogenarians with acute displaced FNF found no differences between HA and THA in terms of hip pain, function, quality of life, reoperations, or ability to perform activities of daily living at two-year follow-up [31]. These findings were corroborated by another large multicenter RCT, the HEALTH trial, which found no difference in the primary endpoint of secondary hip procedures (57/718 for THA compared to 60/723 for HA) within two years of follow-up [32]. The authors also found no differences between groups for mortality and hip instability or dislocation. Although there were moderate improvements in functional outcomes in the THA group compared to the HA group, these differences were considered clinically insignificant based on minimal clinically important difference (MCID) thresholds. Similar results were reported in the long term by Tol et al., who found no differences in mean Harris hip score, revision rate, or mortality at 12-year follow-up of a RCT in patients age 70 or older [37].

Dislocation is often cited as a risk of primary THA vs. HA, with a meta-analysis in 2012 suggesting up to a 2.5-times greater risk of this complication for THA compared to HA [40]. However, conflicting results have been reported in other studies, which is likely due to variations in surgical approach and evolving techniques [23,31]. A more recent meta-analysis supported the finding of increased dislocation rates for THA, but also pointed out that dislocations in HA tended to occur later than in THA, with no significant difference beyond four years of follow-up [35]. Based on this data, the authors suggested that HA should not be chosen solely for the perceived benefit of lower dislocation rates, especially for patients who are expected to live longer than four years.

In the 1970s, the dual mobility cup (DMC) for THA was introduced as an alternative to standard acetabular components in an effort to reduce dislocation rates in THA. The DMC has an additional mobile polyethylene component between the prosthetic head and the outer shell, effectively creating two articulations, which increases stability by increasing the femoral head diameter. Several studies have reported low rates of dislocation for the DMC in primary THA [41,42], as well as revision THA for instability [43,44]. In a systematic review of 54 studies by Darrith et al., the authors found a dislocation rate of 0.46% (41 of 10,783 hips) for primary THAs with DMC, and a dislocation rate of 2.3% (13 of 554 hips) for THA with DMC for treatment of FNF [45]. These results were corroborated by another systematic review of comparative studies by Reina et al., in which the dislocation rate for primary THA with DMC was 0.9% [46]. THA with DMC has also been compared to HA for the treatment of FNF, with the theory that the reduced dislocation risk with DMC would offset the increased risk seen with conventional THA compared to HA—this has been supported by several studies [47,48,49], while other studies show equivocal results [50,51]. Whereas the reports favoring DMC are promising, further prospective, randomized trials with longitudinal follow-up are needed to evaluate long-term results.

Overall, the choice between THA and HA should be tailored to each patient, taking into consideration factors such as baseline mobility, activity level, physiological reserve, and medical comorbidities. With rates of mortality and pain being equivocal, HA is a reasonable option for patients with less demand and who are at higher risk of complications. These patients may benefit from shorter operations with less bleeding and infection risk, although this is balanced by the slightly higher risks of dislocation and subsequent need for revisions. On the other hand, THA may be more advantageous for active patients who have relatively long life expectancy, as they may have higher chances of regaining preinjury function and experience greater benefits in the long term. The option of THA with DMC offers an alternative to conventional THA, with which the risk of dislocation may be significantly decreased.

### 2.5. Bone Cement Implantation Syndrome: Risks and Benefits of a Cemented vs. Uncemented Hemiarthroplasty

**Figure 4:**
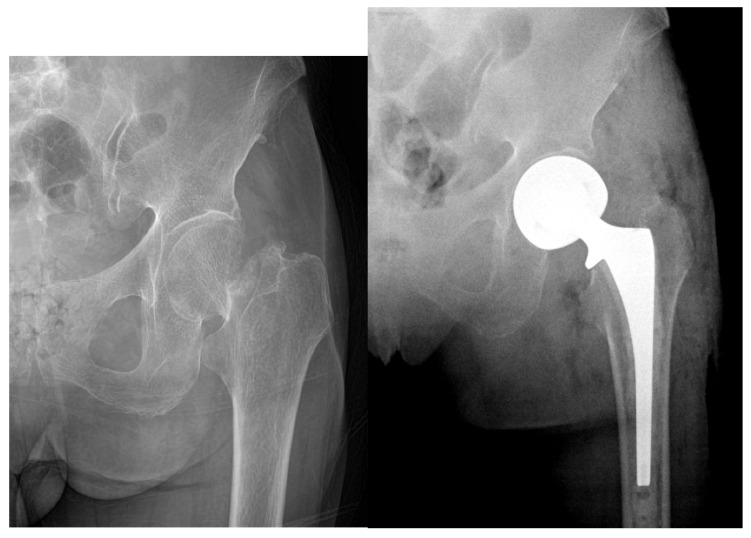
Example radiographs demonstrating uncemented hemiarthroplasty Garden III fracture (Left: Preoperative, right: Postoperative).

Another major topic of debate in the geriatric population is the role of cement in arthroplasty. Current literature supports cemented hemiarthroplasty as the treatment of choice, as it may provide better early functional outcomes, less pain, and decreased risk of intraoperative and postoperative fractures [52,53,54,55,56,57]. Emery et al. studied 53 active patients with displaced FNF who were randomized to either cemented or uncemented hemiarthroplasties and found significantly more hip pain and use of walking aids in the cemented group at mean follow-up of 17 months [54]. Subsequent studies specifically looking at patients older than 65 reinforced the finding of better early- to mid-term function in cemented vs. uncemented arthroplasties, although Barenius et al. found this difference had disappeared by 48 months postoperatively [53,54,55]. Contradicting these results, Langslet et al. reported better functional outcomes via Harris hip scores in uncemented vs. cemented arthroplasties, although they found no difference between groups for quality of life scores or mortality [56]. The increased risk of intraoperative and postoperative periprosthetic fractures after uncemented arthroplasties has also been well documented and cited as a reason to avoid these procedures, especially in patients who have poor tolerance for a secondary operation [53,54,55,56,57] (Figure 4).

A Cochrane review of all RCTs that compared different approaches to arthroplasty for femoral neck fractures described more evidence in support of these findings [58]. Six of the 23 included trials (899 of 2861 total patients) found that cemented prostheses were associated with less pain at one year or greater compared to uncemented prostheses and had a tendency toward better mobility. The authors concluded that there was good evidence supporting more favorable clinical outcomes with cemented prostheses. However, a critique of this review by Evaniew et al. rightly criticized the small sample sizes of many of the included trials, as well as poor follow-up duration, as over half of the included trials had less than 100 participants, with two-thirds of trials reporting less than two years of follow-up [59]. It also highlighted controversy related to nonstandardized early mobilization and aggressive rehabilitation, as the different protocols could have been a confounding factor in correlations drawn between treatment methods and clinical outcomes.

Despite the evidence favoring cemented arthroplasty, recent studies have shown that the majority of arthroplasty procedures in the United States are performed with uncemented components, and global trends indicate an increase in their use over cemented components [60,61,62,63]. This increase was seen even for older age groups, in which cemented arthroplasty has been shown to decrease the risk of revision surgery [63]. This poses a paradoxical situation in which common practice is not reflective of the available evidence. It is unclear why surgeons worldwide are making this choice, but it is likely related to multiple factors, including the historical concern for “cement disease” (which describes osteolysis attributed to the cement, leading to implant loosening and failure), surgeon bias, training practices, and industrial influences [64]. This paradox warrants close monitoring of outcomes and future trends, since the continued use of uncemented arthroplasty may increase the revision burden, as well as future studies investigating the surgeon’s rationale in choosing uncemented arthroplasty.

The use of cement in arthroplasty also raises concern regarding the relatively rare but significant complication known as bone cement implantation syndrome (BCIS). Although there is no standardized definition of BCIS, it is generally characterized by a collection of cardiopulmonary symptoms including hypoxia, hypotension, arrhythmia, and cardiac arrest associated with fat and bone marrow embolization that may occur during femoral reaming and cementation [65]. These symptoms can also result in sudden death both intraoperatively and postoperatively. Due to the spectrum of symptoms that encompass BCIS, the true incidence of this syndrome is difficult to identify. Donaldson et al. evaluated three series studying BCIS and concluded that the incidence of intraoperative mortality during cemented THA was 0.11%, with no corresponding intraoperative deaths in the associated uncemented THA or hemiarthroplasty cohorts [65]. The largest of these series found that cemented arthroplasty for fracture diagnosis placed patients at greater risk for intraoperative death compared to elective arthroplasty [66]. Other factors that increased mortality risk included older age, poor preexisting physical reserve, impaired cardiopulmonary function, and metastatic disease. Most recently, Ekman et al. conducted a retrospective review of 3010 patients who received cemented vs. uncemented HA for FNF to evaluate mortality at multiple time points [67]. Although the raw data showed more deaths for cemented HA (50/1935 vs. 22/1173 for uncemented HA) during the first two days postoperatively, the difference in mortality was not statistically different once adjusted for age, sex, ASA class, and year of surgery. This association did not change for any other time point. Subgroup analyses for patients who were ASA class IV, indicating severe systemic disease, showed a difference in mortality for the cemented HA group, although not statistically significant, during the first two days postoperatively. The authors concluded that cemented HA may be a safe option, but high-risk patients may need more cautious monitoring, especially intraoperatively during cementing.

There are several surgical strategies that have been shown to reduce the risk of BCIS, most of which involve decreasing the formation and push of emboli into the circulation [68]. Vacuum mixing of cement reduces its porosity, which, in turn, helps reduce air emboli that are formed during cementation, and retrograde insertion of cement into the femoral canal helps reduce intramedullary pressures. Performing medullary lavage prior to cementation and prosthesis insertion has also been shown to decrease the number of emboli formed. If BCIS is suspected, prompt treatment with aggressive fluid resuscitation and inotropic agents to augment right heart function should be initiated. Pulmonary vasodilators may also be used to treat the increase pulmonary vascular resistance triggered by emboli-related mediators. 

Despite the rarity of BCIS, the immediate and long-term sequelae can be severe, and every measure should be taken to reduce the risk of its occurrence. It is especially imperative for surgeons and anesthesiologists to communicate effectively in the operating room in order to expedite treatment should the need arise.

## 3. Discussion/Conclusions

The treatment of FNF remains an important topic of investigation, as these injuries are associated with high rates of morbidity and mortality, especially in the geriatric population. Our review of the current literature highlights the different risks and benefits associated with the three main treatment options for FNF: Internal fixation, hemiarthroplasty, and total hip arthroplasty.

There is consistent evidence to support the use of internal fixation, either with cannulated screws or sliding hip screws, for the treatment of most patients with nondisplaced FNF. These methods generally result in good outcomes in terms of pain and hip function. However, given that internal fixation also comes with the risk of higher rates of reoperation, arthroplasty may be considered in patients who may not tolerate secondary procedures.

For displaced FNF in the elderly, earlier controversy regarding the use of internal fixation vs. arthroplasty has now mainly been resolved, with the evidence pointing toward the superiority of arthroplasty in the majority of these fractures. However, the choice between HA and THA and the role of cement remains uncertain. THA may be preferred for high-demand patients who can benefit from improved postoperative function and longevity of the implant, while hemiarthroplasty may be sufficient for patients with many comorbidities who are unable to tolerate longer operations or the risk of reoperation. In addition, the use of THA with DMC may help offset the disadvantage of dislocation seen with conventional THA, although further studies are needed to evaluate the superiority of THA with DMC compared to HA. Despite the lack of definitive evidence, the use of THA has increased in many parts of the world.

Despite evidence to support better functional outcomes and decreased risk of revision surgery with cemented compared to uncemented arthroplasty, the worldwide use of uncemented components has increased. The reasons behind these trends warrant further investigation. Cemented arthroplasty comes with the additional risk of BCIS, which is a devastating but rare complication, and recent evidence has shown that there is no significant increase in mortality with cemented arthroplasty except for possibly in the frailest and highest risk patient group. When caught early, BCIS can be treated with aggressive fluid resuscitation, inotropic agents, and pulmonary vasodilators.

Future studies in FNF treatment will likely be geared toward elucidating optimal factors for arthroplasty, including differences in surgical approach and further long-term studies on the use of cement. Striking a balance between the advantages and disadvantages of each treatment option is difficult, and it is imperative for physicians to carefully consider each individual patient’s health profile to aid in selecting the optimal treatment method.
